# Posttransplant Lymphoproliferative Disease after Lung Transplantation

**DOI:** 10.1155/2013/430209

**Published:** 2013-03-05

**Authors:** Isabel P. Neuringer

**Affiliations:** Pulmonary and Critical Care Unit, Massachusetts General Hospital, Bullfinch 148, 55 Fruit Street, Boston, MA 02114, USA

## Abstract

Posttransplant lymphoproliferative disease (PTLD) after lung transplantation occurs due to immunosuppressant therapy which limits antiviral host immunity and permits Epstein-Barr viral (EBV) replication and transformation of B cells. Mechanistically, EBV survives due to latency, escape from cytotoxic T cell responses, and downregulation of host immunity to EBV. Clinical presentation of EBV may occur within the lung allograft early posttransplantation or later onset which is more likely to be disseminated. Improvements in monitoring through EBV viral load have provided a means of earlier detection; yet, sensitivity and specificity of EBV load monitoring after lung transplantation may require further optimization. Once PTLD develops, staging and tissue diagnosis are essential to appropriate histopathological classification, prognosis, and guidance for therapy. The overall paradigm to treat PTLD has evolved over the past several years and depends upon assessment of risk such as EBV-naïve status, clinical presentation, and stage and sites of disease. In general, clinical practice involves reduction in immunosuppression, anti-CD20 biologic therapy, and/or use of plasma cell inhibition, followed by chemotherapy for refractory PTLD. This paper focuses upon the immunobiology of EBV and PTLD, as well as the clinical presentation, diagnosis, prognosis, and emerging treatments for PTLD after lung transplantation.

## 1. Introduction

After lung transplantation, the allograft recipient is typically prescribed immunosuppressant therapy to inhibit adaptive immunity and cellular rejection, thus concurrently limiting innate and antiviral host response. The presence of Epstein Barr virus (EBV) affects 90% of the world's population, with immunity to EBV present in the majority of adults [1], and thus the majority of donor organs are EBV positive [2]. Those who are EBV naïve at the time of transplant are more likely to acquire an infection from the donor and progress through primary EBV infection to viral transformation of naïve B cells, resulting in posttransplant lymphoproliferative disease (PTLD) [3]. Conditions such as plasma cell hyperplasia and primary EBV infection may be viewed as manifestations of potential PTLD, while polymorphic PTLD, monomorphic PTLD, B cell neoplasms, T cell neoplasms, and classical Hodgkin lymphoma constitute neoplastic processes as recently reviewed [4]. Due to higher levels of immunosuppression in thoracic organ transplantation compared to recipients of other solid organs with the exception of intestinal transplantation, the rate of PTLD in lung transplant recipients may range between 5% and 15% [5–10]. In one of the first reports of PTLD after lung transplantation over 20 years ago, Armitage and colleagues reported an incidence of 7.9% of PTLD, observing a peak occurrence of PTLD within the first year and different clinical outcomes for PTLD occurring after 1 year. Early PTLD responded to reduced immunosuppression, was disseminated in a minority of cases, and carried a mortality of 36%, while late-occurring disease did not respond to lowered immunosuppression, was disseminated at presentation, and had a mortality of 70%. In this early study, the majority of cases of PTLD also followed primary EBV infection [5]. Reports from lung transplant centers over a more recent era of 10 years have demonstrated an incidence of ~5% [11–13], the reduced rate attributed to a combination of factors such as prolonged antiviral prophylaxis, improved detection assays, and anticipatory surveillance of EBV negative patients. Nevertheless, despite advances in earlier detection of DNA viremia, after lung transplant care, and immunomodulatory therapies, the mortality still may approach greater than 50% due to infectious complications or late-presenting or refractory PTLD. This paper will discuss current advances in understanding EBV immunobiology, risk factors for PTLD, clinical presentation, diagnosis, staging, and therapies.

## 2. Immunobiology of EBV and PTLD

In general, PTLD occurs after EBV infection of the nasopharynx and epithelial cells, with subsequent B cell transformation due to altered host immunity after transplantation. Initial EBV infection results in a cellular program aimed at viral production through ultimately lytic infection of tonsillar B cells and establishment of a latent infection which is a life-long one. During type III latency, a period of growth and survival of infected B cells, genes expressed include Epstein Barr nuclear antigens (EBNAs 1-3C), latent membrane proteins (LMPs 1-2B), and nuclear RNAs (EBERs). Subsequently, during type II latency, LMP 1 and LMP 2 provide differentiation within germinal centers, through CD40, a common pathway for T helper signaling of B cells. Lastly, during type I latency, no genes are expressed, thus evading cytotoxic T cell responses [14]. Latent proteins which promote B cell immortalization include EBNA-1, EBNA-2, EBNA-3A, EBNA-3C, EBNA-LP, and LMP-1. Functions associated with these proteins include replication of the EBV genome, upregulation of c-myc, cell cycle checkpoint inhibition, binding of CD40, anti-apoptosis pathways through Bcl-2, and modulation of intracellular signaling pathways including NF*κ*B [15].

**Figure 1:**
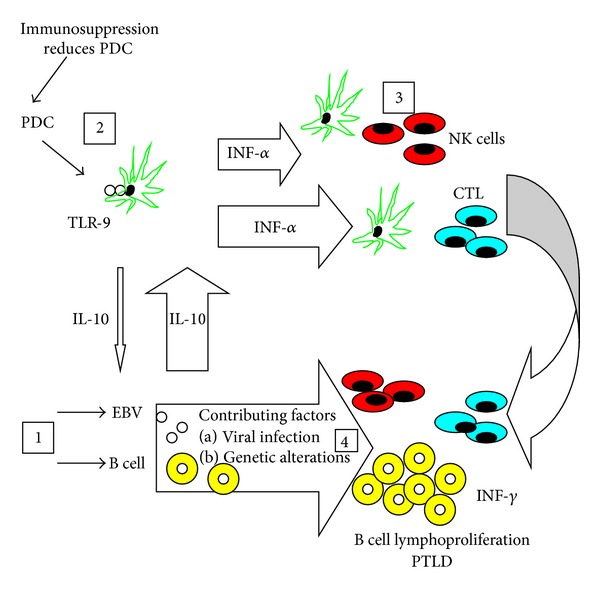
This proposed model of pathogenesis shows EBV infection of B cells (1), concurrent immunosuppression of plasmacytoid dendritic cells, PDCs, (2), net increase in IL-10, release of *α*-interferon and *γ*-interferon, and stimulation of cytotoxic T lymphocytes and NK cells (3), and B cell lymphoproliferation (4). From [16].

Host immunity to EBV is inhibited by EBV-derived cytokines which downregulate cytotoxic T cell responses and induction of anti-apoptosis pathways which prevent cell death by EBV latency proteins. During lytic infection, viral interleukin-10 (vIL-10), bearing homology to human IL-10, reduces IL-12 and *γ*-interferon release essential for cytotoxic T cell activity. Similarly, an EBV-secreted soluble receptor causes inhibition of colony-stimulating factor-1 (CSF-1) necessary for monocyte-associated antiviral cytokine production. Stimulation of anti-apoptosis results from blockade of death receptor signals at the cell surface (Fas, TNF-related apoptosis inducing ligand), Bcl-2 amplification within mitochondria (intrinsic pathway), and NF*κ*B activation within the cell. Please see Figure 1 for a diagram of EBV proliferation.

Antiviral responses are predominantly mediated by cytotoxic T cells, primed by prior immunological stimuli, with specific antigen-specific memory. Mifsud and colleagues described EBV-specific CD8+ T cells in a longitudinal study cohort of lung transplant recipients, focusing upon an HLA-B8 restricted cohort with reactivity directed towards epitope FLRGRAYGL on the EBV protein EBNA3A. The authors found the frequency of EBV-specific T cells in immunosuppressed lung transplant patients to be 4-5-fold greater compared to healthy controls. Although ex vivo stimulation of EBV specific T cells revealed alloreactive responses, *γ*-interferon production was blunted and variable among the patient's peripheral T cells tested, compared to healthy controls, suggesting limited cytotoxic responses in EBV seropositive lung transplant patients likely persisting after initial EBV infection [17]. In a pediatric thoracic transplant population, children with high EBV viral loads demonstrated significantly higher number of CD8+ T cells and EBV-specific CD8+ cells compared to those with undetectable viral loads, although they showed no difference compared to healthy controls. Examination of *γ*-interferon production as related to level of EBV viral load showed that those children with high viral loads had significantly lower levels of *γ*-interferon production compared to those with low viral loads, consistent with depletion and reflecting a possible mechanism for the 45% rate of progression to PTLD in these patients [18]. In addition to immunological exhaustion demonstrated by these EBV-specific T cells, a subsequent study of pediatric thoracic transplant recipients with PTLD by Wiesmayr and coworkers also identified impaired NK cell responses, as evidenced by decreased expression of NKp46 and NKG2D and increased PD-1 expression, thus limiting T cell responses [19]. Taken together, these studies provide significant insight into pathogenic mechanisms which amplify EBV-infected B cell immortalization, constrain maximal host immunity, and facilitate PTLD.

## 3. Clinical Presentation of PTLD after Lung Transplantation

### 3.1. Risk Factors

Identifiable risk factors for PTLD in lung and solid organ transplantation may be related to multiple factors unique to the type of organ transplanted, as the rate of PTLD increases from the lowest in kidney, intermediate for pancreas, liver, heart, and lung, to highest in small intestine transplantation [20]. Early presentation within the first year after lung transplantation is associated with primary EBV infection in a seronegative, naïve patient [6, 21, 22] and pediatric thoracic transplant recipients [23, 24], underlying cystic fibrosis diagnosis likely due to EBV seronegative status [6, 21], primary CMV infection [4], higher rejection frequency [25], and use of induction therapy at time of transplant [4, 25, 26]. In general, later onset PTLD was associated with extent of exposure to higher levels of immunosuppression, older recipient age, and male gender [4]. Individual lung transplant centers have also reported PTLD which was not associated with pretransplant EBV serological status or occurred more frequently in recipients who were EBV positive before transplant. These centers identified PTLD predominantly in older patients with an underlying diagnosis of COPD, as well as equally distributed between those with COPD, IPF, Eisenmengers, and alpha-1 antitrypsin deficiency [9, 11]. Immunosuppressant regimens may impact upon the development of PTLD, as recent reports from kidney transplantation cite an increased risk of PTLD in patients receiving mTOR inhibitors and tacrolimus compared to patients receiving cyclosporin and mycophenolate mofetil [27]. To date, few studies in the lung transplant literature have cited clear PTLD risk with distinct immunosuppressant regimens, other than overall immunosuppressant load and use of induction therapy. Lastly, Wheless and coworkers identified an association between HLA-A3 allele either of donor or recipient status and an increased incidence of PTLD in lung transplant recipients [3].

### 3.2. Monitoring EBV Viral Load

Early means of testing for primary EBV infection and the immunological program associated with current or past infection relied upon serological sampling and an understanding of the phases of EBV infection. Sequentially, IgM antibodies to EBV viral capsid and early antigen rise and fall within the first month, followed by antibodies to EBNA and the sustained IgG antibody response to viral capsid antigen [28]. Monitoring of the EBV DNA load by quantitative PCR has replaced serological testing, although variability exists for techniques for DNA extraction, optimal targeted sequence, and ranges of detection by commercial assays and custom-designed institutional primer pairs [29–32]. Thus, clinicians have relied upon initial assessment of EBV status before transplant and serial testing aimed at assessing trends in viral load, rather than a single, on-time value. There have been few studies addressing the sensitivity and specificity of EBV viral load testing in lung transplantation, and given the limited numbers of patients, they have yielded variable results. In a study of 13 cases of PTLD after lung transplantation, 5 of which occurred during the recent era of viral load monitoring, EBV viral load levels at the time of detection were significantly higher in those who were seronegative before transplant. The 1 patient who was seropositive before transplant exhibited an elevated EBV viral load when diagnosed with PTLD. The 4 patients who were seronegative before transplant all had detectable levels, but only 2/4 patients' were elevated, conferring a sensitivity of 50% and specificity of 22% [3]. Tsai and colleagues examined a panel of EBV proteins expressed in lung transplant patients (group 1) and those undergoing evaluation for PTLD (group 2). In group 1, a minority had positive EBV viral load detected in plasma, and ~40% had intracellular EBV. In group 2, of whom ~50 had PTLD, approximately 90% had detectable EBV by PCR and thus demonstrated high sensitivity for EBV-positive PTLD. Testing for LMP-1, EBER-1, and EBNA-1 together conferred a sensitivity of 92% and specificity of 72%, with plasma EBNA PCR yielding the greatest sensitivity (77%) and specificity (100%) [33].

### 3.3. Clinical Presentation

Recognition of predisposing risk factors for PTLD and the routine testing of EBV viral load after lung transplantation have contributed towards earlier diagnosis and have likely altered overall mortality. The clinical presentation of PTLD may also depend upon time from transplantation to diagnosis of PTLD, with earlier disease occurring within the first year more likely to present within the thorax including allograft parenchyma or mediastinal lymph nodes [6, 9, 11–13, 34]. After the first year after transplant, extrathoracic presentation including the gastrointestinal tract is a common site of PTLD due to the high density of lymphoid tissue [9, 11, 13]. Other sites reported include cervical lymph nodes [35], central nervous system including epidural mass with spinal cord compression [36], bone marrow [11], and paranasal sinuses [37].

Clinical symptoms of PTLD relate to the systemic effects of lymphoproliferation and may include lymph gland tenderness, fever and sweats, fatigue, sinus pain, cough, headache, intestinal pain, and neurological deficits. Examination of patients with PTLD may reveal enlarged lymph nodes or nodules, tonsillar prominence, hepatosplenomegaly, intestinal obstruction, and neurologic deficits.

### 3.4. Laboratory Testing

Since PTLD presents with systemic manifestations, laboratory testing may provide additional insight into localization of PTLD. Testing of complete CBC, chemistries, liver function tests, LDH, uric acid, and hemoccult positivity may reveal systemic anemia, involvement of the reticuloendothelial system of the liver and spleen, tumor lysis from rapid cell growth and turnover, and GI bleeding from neoplastic lesions. Concurrent viral infection furthers host immunosuppression and may be a cofactor in PTLD tumor growth. Thus, assessment of quantitative CMV viral load should be routinely performed. EBV serology is an insensitive and nonspecific test for PTLD due to altered host antibody responses and may be difficult to interpret as well after transfusion of red blood cell or plasma products. Quantitative measurement of EBV viral load used for surveillance monitoring and indication of systemic disease may be limited by insensitivity and lack of specificity but, nevertheless, remain consistent within individual laboratory testing [4]. Michelson and coworkers analyzed bronchoalveolar lavage (BAL) fluid from 16 heart-lung transplant patients for quantitative PCR for EBV and found values 50 times higher in patients with PTLD. BAL proved to be highly sensitive in detecting 3/3 cases of PTLD compared to testing of peripheral blood [38].

### 3.5. Radiology and Imaging of PTLD

Since PTLD is likely to present within the thorax, chest X-ray (CXR) may reveal parenchymal lesions, infiltrates, and nodules, followed by CT scan of the chest, abdomen, and pelvis, to assess lymphadenopathy. Concurrent imaging of the head and neck may be performed given PTLD predilection for the CNS and cervical lymph nodes. In recipients of solid organ transplant, pulmonary nodules as noted on CXR and CT scanning in EBV seronegative patients and lung transplant recipients proved to be associated with PTLD (odds ratios 21.7 and 36.6, resp.) [39]. Fluoro-2-deoxy-D-glucose (FDG-PET) has become essential for accurate staging of extranodal and extrathoracic sites of disease, as reported by Marom and colleagues [40]. In a retrospective analysis of lung transplant patients who had developed PTLD, 4 patients were identified who underwent FDG-PET, accompanied by CT scanning. All pulmonary nodules noted on chest CT >5 mm in diameter were identified by FDG-PET. A paracardiac lymph node and pleural thickening were FDG-PET avid, as were low-attenuation lesions on CT in the liver and areas of bowel thickening. FDG-PET also detected areas not noted on CT scanning, such as abdominal and axillary lymph nodes. In this study, FDG-PET was used to monitor response to therapy as well. FDG-PET has been proven superior to CT or PET alone with improved sensitivity and specificity for PTLD after solid organ transplantation [41, 42].

In terms of localization of PTLD, intrathoracic location is present in >70% of lung transplant recipients [6, 9, 11–13, 34], and radiological findings provided the first sign of disease. In 50% of cases of intrathoracic PTLD, a single pulmonary nodule was most frequently found. Additional abnormalities included numerous nodules, infiltrates, and thoracic adenopathy. Importantly, a single pulmonary nodule was associated with a better overall prognosis, as 8/9 patients with intrathoracic PTLD were alive 1 year after diagnosis [43]. Radiographically, GI involvement has been detected in 20% of lung transplant recipients, most often occurring in the distal small bowel, and may reveal bowel wall thickening or mass lesions [44, 45]. Involvement of the liver with discrete nodularity [9] and CNS mass lesions have also been noted on imaging [46]. Complete staging may therefore be accomplished by full body imaging, assessment of bone marrow, and CNS.

### 3.6. WHO Classification, Histopathology, and Staging of PTLD after Lung Transplantation

Current standard diagnosis of PTLD requires a tissue diagnosis, evaluation of histopathological morphology, immunophenotype, presence of clonality, and testing of tissue for EBV using in situ hybridization for EBER. Categories of PTLD as categorized by the WHO classification of tumors of hematopoietic and lymphoid tissues list early lesions, polymorphic PTLD, monomorphic PTLD including B and T cell tumors, and classical Hodgkins disease. Early lesions are considered benign and include infectious mononucleosis-like and plasmacytic hyperplasia. Polymorphic PTLD demonstrates mature lymphocytes and polyclonal or monoclonal B cells for the majority, while monomorphic lesions are comprised of clonal B lymphocytes. B cell neoplasms may include diffuse large B cell lymphoma (DLBCL), Burkitt's lymphoma, plasma cell myeloma, and plasmacytoma-like lesion, while T cell neoplasms may include peripheral T cell lymphoma and hepatosplenic T cell lymphoma [47]. The two most commonly found histopathologies of PTLD after lung transplantation have been monomorphic diffuse large B cell lymphoma (DLBCL) [11, 12, 48, 49] and polymorphic B cell lymphoma [3, 9], with polymorphic predominating in early PTLD, while monomorphic characteristic of later onset [13].

Recent consensus statements conclude that classification according to WHO categories describes histopathological features alone and therefore requires a complement of additional studies to characterize immunohistochemistry, testing for CD20 positivity, clonality, and in situ studies demonstrating a clear EBV-related neoplasm, as related to the clinical context such local or disseminated disease [4, 50]. Glotz and colleagues have also recommended addition of a prognostic index, such as the International Prognostic Index [50]. The International Prognostic Index (IPI) is composed of 5 factors which may segregate rapidly growing lymphomas into different survival outcomes based upon the number of negative prognostic factors. These factors include age >60, stage of tumor (grade III/IV disease), performance status, extranodal disease, and elevated serum lactate dehydrogenase (LDH). A low or intermediate risk group would therefore have age <60, stage I or II, lymphoma located within the lymph nodes, intact functionality, and normal serum LDH [51].

In addition to histopathology and ancillary testing to provide insight into subsequent focused therapy, advances in molecular and genetic analyses define additional features of PTLD. As reviewed by Ibrahim and colleagues, distinct genetic rearrangement, amplification, and somatic hypermutation involving point mutations of the immunoglobulin (Ig) variable region are associated with BCL6, BCL2, p53, and PAX5 genes in PTLD. Gain or loss of chromosomal material is frequently identified in PTLD such as DLBCL, for example, a gain of chromosomes 5p and 11p and deletions of 12p, 4p, 4q, 12q, 17p, and 18q.

Deletions of 4q, 17q, and Xp are unique to PTLD as contrasted to lymphomas in immunocompetent patients. Interestingly, in PTLD-related DLBCLs, a lack of del(13q14.3) which is hypothesized to play a role in immune surveillance avoidance may reflect genetic alterations in immunosuppressed patients [16]. Lastly, DNA hypermethylation of tumor suppressor genes, apoptotic pathways, and genes involved in cell cycle regulation can be identified in PTLD. Such genes found to be hypermethylated include death-associated protein kinase (DAP-k) which plays a role in apoptosis, O6-methylguanine-DNA methyltransferase (MGMT), a DNA repair gene, P73, a tumor suppressor gene, P16, a cell cycle inhibitor, and SHP1 gene, a cell cycle regulator [16].

### 3.7. Prognosis

In general, poor performance status, wide-spread disease, CNS involvement, monoclonality, and T-, NK-, or EBV-negative PTLD are associated with less favorable outcome [4]. Early PTLD as opposed to later onset PTLD may confer differences in survival outcomes, as earlier lesions may be more amenable to reduced immunosuppression. A retrospective review of all cases of PTLD at the Mayo Clinic (Rochester) further noted that there were differences between PTLD manifested early compared to disease onset at a relatively late time point after transplant. The investigators found that positive EBV in situ hybridization status, CD20-positive status, and involvement of the organ that was transplanted were associated with early presentation, however, did not find differences in outcomes between PTLD presented early compared to later [52]. Paranjothi and colleagues from Washington University in St. Louis Lung Transplant program confirmed the majority of PTLDs localized within the thorax during the first year after lung transplantation and that subsequent survival was better in those patients exhibiting involvement of the allograft in PTLD compared to those with extra-thoracic disease [8].

Whether PTLD results from donor or recipient origin may also play a role in prognosis, as Olagne and colleagues documented in a large cohort of kidney transplant patients, noting survival of 68% at 5 years in PTLD of recipient origin, compared to 85% survival in PTLD of donor origin, although this did not attain statistical significance [53]. The majority of PTLDs result from transformation of recipient lymphocytes and are thus of recipient origin [54]; however, some PTLDs are due to donor cells transmitted with the organ or associated lymphoid tissue during transplantation [55]. There is limited data on percentages of PTLD of recipient or donor origin in the majority of studies involving lung transplantation yet is likely to play a role in outcomes and management of host immunosuppression.

Data from individual lung transplant centers' experiences cite a mortality figure of 30%–60% due to PTLD [5, 6, 9, 10, 12, 48]. A recent individual center report of 32 patients with PTLD from the University of Minnesota Lung Transplant program cited an overall mortality of 75%, including 11/32 due to infection associated with PTLD and chemotherapy, 9/32 from PTLD, and 4/32 from other causes not associated with PTLD [11]. Another recent report from the University of Pennsylvania Lung Transplant center found that patients transplanted between the years 2000 and 2011 had better survival than those transplanted between the years 1990 and 1999 [13]. Taken together, these data confirm that PTLD still remains a significant cause of morbidity and mortality after lung transplantation. Please refer to Table 1 for a summary of key clinical findings, histopathology, and treatment of PTLD in lung transplant patients at individual transplant centers.

### 3.8. Prevention and Monitoring of EBV Viral Load

Essential steps in preventing PTLD include a combination of strategies, including identification of high-risk EBV-naïve patients and routine surveillance for EBV viral load, antiviral prophylaxis, and reduction in immunosuppression in appropriate high-risk patients who have seroconverted their EBV status and have detectable and/or rising EBV quantitative PCR. Prior to transplantation at the time of evaluation, patients at risk for primary EBV or CMV infection should be identified, and appropriate serological testing should be performed. After transplantation, patients whose immunosuppression is being escalated, or who have received T cell depleting therapies, should be monitored as well. Antiviral prophylaxis is routinely given after lung transplantation, primarily for HSV and CMV, but likely has added chemoprophylaxis for EBV. Malouf and colleagues described a significantly diminished rate of PTLD in lung transplant recipients after elimination of induction therapy and institution of viral prophylaxis in EBV seronegative patients, decreasing from 4.2% to 0.76% [56]. Ganciclovir was shown to be superior to acyclovir for reducing the odds of developing PTLD in a renal transplant population, as prophylactic antiviral use was associated with up to 83% reduction in the risk of PTLD [57]. The addition of intravenous immunoglobulin (IVIg) to ganciclovir therapy did not reduce EBV viral loads compared to the use of ganciclovir alone [58]. Current practices of prolonged CMV prophylaxis after lung transplantation [59] may cause an unintended but beneficial effect upon reducing EBV seroconversion during the early, highest period of immunosuppression after transplant.

Although limited data exists in lung transplantation regarding the prophylactic reduction of immunosuppression in patients with presumptive PTLD, studies have shown that under unique circumstances, immunosuppression may be reduced safely without risk for acute or chronic allograft dysfunction. Bakker and colleagues followed EBV viral loads in a cohort of lung transplant patients on average 4 years posttransplant, and reduced immunosuppression after finding evidence of EBV reactivation, with no acute rejection, acceleration of BOS, or poorer survival. The authors hypothesized that EBV viral loads in this setting late after transplant indicated the net state of immunosuppression [61]. Although current practice involves reduction in immunosuppression in the face of a rising EBV viral load and suspicion of PTLD, there is limited data regarding incidence of subsequent acute rejection and BOS when PTLD occurs within the first year after lung transplantation, and immunosuppression is reduced.

### 3.9. Treatment

**Figure 2:**
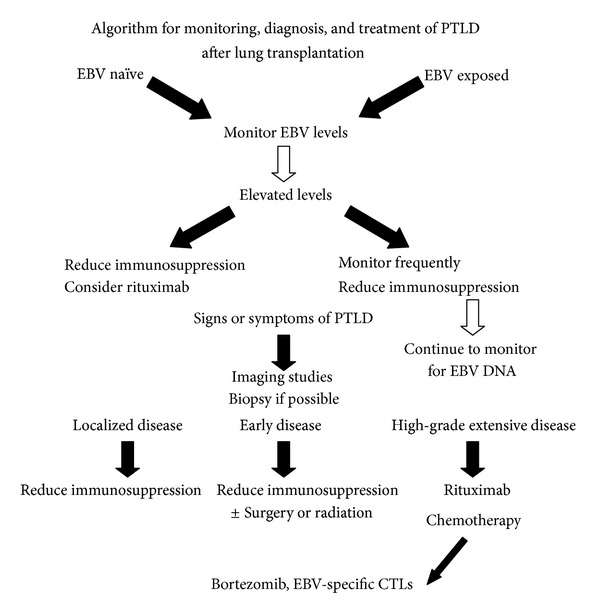
This algorithm proposes routine surveillance of high-risk patients to enable diagnosis at an early stage. Starting with EBV viral load monitoring, patients who manifest elevated levels with symptoms would progress to imaging studies and biopsy of enlarged lymph nodes or nodules. Identification of CD20 lesion positivity, cytogenetics, immunostaining for LMP and EBER, and assessment of monoclonal or polyclonal proliferation can focus further therapy. In localized disease, reduction of immunosuppression or surgery may be sufficient to control disease, while rituximab may be given concurrently. High-grade extensive disease may require chemotherapy, bortezomib, or EBV-specific cytotoxic T lymphocytes if available. From [60].

The overall approach to treatment of PTLD will vary according to the individual risk, clinical presentation, and stage and sites of disease, as depicted in Figure 2. In general, treatment starts with reduction of immunosuppression, antiviral therapy if early in pathogenesis, anti-CD20 (monoclonal B cell inhibitor), and chemotherapy with CHOP for more widely disseminated disease. For early presenting EBV-positive PTLD, immunosuppression is reduced specifically by elimination of the cell cycle inhibitor/antimetabolite, decreasing calcineurin inhibition (CNI) and lowering corticosteroid dosing. Practices from individual lung transplant centers have reported reduction in immunosuppression, antiviral therapy, surgical intervention for large masses and/or abdominal perforation, rituximab, and lastly chemotherapy for refractory or late-staged cancers [5, 6, 8–13]. For localized or bulky disease within the chest, as well as colonic obstruction, surgery may play a role in debulking disease or averting colonic perforation. Rituximab has become an effective agent directed against CD20-positive B cell tumor cells and has been proposed as a first-line agent against PTLD. This murine/human monoclonal antibody blocks steps in cell cycle progression and differentiation, through complement and antibody-mediated cytotoxicity. After Cook and coworkers cited a 66% response rate to rituximab in lung transplant recipients with PTLD, additional investigators have subsequently applied this approach in solid organ transplantation [62]. Knoop and coworkers achieved clinical remission in 66% of lung transplant patients with PTLD after 4 courses, with a median survival subsequently of 34 months [35]. Oertel and colleagues achieved 52% remission in recipients of solid organ transplant including lung, who had developed PTLD, and noted a survival of 37 months after PTLD [63]. Similar results have been reported from other centers, with clinical remission rates of ~60% [64–66]. Nevertheless, long-term survival may still be impacted, studies are difficult to interpret due to uncontrolled treatment protocols, and timing and dosing period of therapy have yet to be determined. Common side-effects reported include immediate hypersensitivity to the infusion, nausea, vomiting, fever and chills, systemic effects of tumor degradation, neutropenia, and profound B lymphocyte depletion with increased susceptibility for CMV reactivation. Another therapy targeting B cell lymphoma cells is a compound called bortezomib, a boron-based drug which causes proteosome inhibition. A clinical trial using bortezomib for EBV-positive PTLD in conjunction with rituximab is underway and may prove to be more effective than rituximab alone (ClinicalTrials.gov, no. NCT01058239).

 Chemotherapy may be required for patients with disseminated disease, clinical progression on rituximab, or non-B cell PTLD. Regimens used by individual lung transplant centers include a combination of the following chemotherapeutic agents: cyclophosphamide, adriamycin, vincristine, etoposide, and prednisone [5, 6, 8–13]. In a clinical trial of rituximab followed by chemotherapy for PTLD (ClinicalTrials.gov, no. NCT01458548), 60% of solid organ patients had clinical remission and extended survival on average of 6.6 years [67]. Complications of systemic chemotherapy are well documented and include nausea, vomiting, anemia, neutropenia, lung injury, infections, and sepsis [4].

Promising therapies are emerging in the treatment of PTLD, such as EBV-specific cytotoxic T cell therapy, which as yet is still in experimental phase. Cytotoxic T lymphocytes (CTLs) from healthy, EBV-experienced donors are adoptively transferred in order to better respond to EBV-positive tumor cells in immunosuppressed solid organ transplant patients. Optimally, HLA-matched allo-CTLs provide better results and may be stored in a frozen bank after being obtained from donors. This therapy was applied to solid organ transplant patients with advanced PTLD in a phase II clinical trial and resulted in ~80% survival at 6 months [68, 69]. In a study of 3 patients who received allo-CTLs, a lung transplant patient with monomorphic B cell PTLD who had failed reduced immunosuppression, rituximab, and chemotherapy received allo-CTLs with epitope specificity to EBNA1, however, succumbed to respiratory failure. The investigators identified tumor infiltration by the allo-CTLs, thus demonstrating appropriate engagement of target cells, but inadequate control of the adoptive response [59]. Further optimization of allo-CTL therapy in terms of dosing, epitope specificity, HLA match, and recipient functional status may help apply this therapy to the clinical arena.

 Lastly, patients who survive PTLD and have chronic lung allograft dysfuntion or experienced allograft dysfunction due to drug toxicity, may be considerd for retransplantation. In a review of solid organ transplant recipients who survived PTLD and underwent retransplant, time from PTLD to retransplant was greater than 1 year in 75% of patients, with median number of days 862 in the 9 lung transplant recipients who were part of the analysis. The majority of lung transplant patients in this analysis were pediatric. At the time of analysis, 55.6% of the lung retransplant patients were alive with a mean followup of 776 ± 249 days [70].

As collective experience grows with management of PTLD after lung transplantation, optimal care, monitoring of high-risk recipients, and treatment continue to be refined. Additional monitoring tools may include soluble CD30 which has been associated with lymphoproliferative disorders [71] and quantification of circulating antibody free light chains associated with B cell dysfunction prior to and during PTLD [72]. Expansion of EBNA1-specific CD4+ T cells may improve the feasibility of adoptive immunotherapy, ultimately for autologous use [73]. Thus, despite continued challenges in managing PTLD, significant progress has been made in risk stratification of lung transplant recipients, monitoring and earlier diagnosis of PTLD, and biologically directed cell-specific therapies, which will continue to improve outcomes and survival.

## Figures and Tables

**Table 1:** Summary of outcomes of lung transplant patients with PTLD from US and European lung transplant centers.

Study/year/ reference	#PTLD total	PTLD incidence	Time to Dx PTLD after transplant	Sites of presentation	Pathology	Treatment	Survival
Armitage et al. 1991, [5]	5/87	7.9%	4 months	Lung, mediastinum, GI tract	NA	RI, surgical resection, CH	36% mortality <1 year, 70% mortality >1 year
Aris et al. 1996, [6]	6/94	6.4%	4.5 months	Mediastinal mass, tonsil enlargement, lung nodules, bowel obstruction, skin nodules	Polymorphic B cell hyperplasia and lymphoma, monoclonal	RI, surgical excision of mediastinal mass, CH	Average survival ~11 months
Wigle et al. 2001, [10]	12/242	5.0%	17.6 months	Lung, mediastinum, abdomen	NA	RI, CH	1 year survival rate of 58%
Paranjothi et al. 2001, [8]	30/494	6.1%	402 days	Lung, mediastinal lymph nodes, liver, testicle, GI tract, bone marrow, skin, ovary	Monomorphic lymphoma, polymorphic lymphoma	RI, surgical resection, rituximab, CH	Median survival 1.0 ± 1.5 years
Ramalingam et al. 2002, [48]	8/244	3.3%	12 months	Lung, small bowel, colon, skin	Monomorphic lymphoma, polymorphic lymphoma	RI, rituximab, interferon, CH, XRT	5/8 patients died of complications from PTLD
Reams et al. 2003, [9]	10/400	2.5%	343 days	Lung, small bowel, liver, periaortic adenopathy, tongue, supraclavicular	Monomorphic lymphoma, polymorphic lymphoma, T cell lymphoma	RI, rituximab, surgical resection, CH, XRT	5/10 survival over 1992–2002 time period
Tsai et al. 2008, [33]	17/206	8.3%	NA	Lung, extranodal	Monomorphic lymphoma	RI, rituximab, surgical resection, CH, XRT	Median survival 12 months
Knoop et al. 2006, [35]	17/224 *6/17	8.0%	68 months	Lung, mediastinum, cervical nodes, liver, bone marrow	Monomorphic lymphoma	RI, rituximab	4/6 complete remission with relapse free survival of 34 months
Baldanti et al. 2011, [12]	5/111	4.5%	270 days	Lung, mediastinum	Hodgkins, monomorphic lymphoma	RI, rituximab, CH	4/5 have died
Wudhikarn et al. 2010, [11]	29/639	5.0%	40 months	Lung, GI tract, intraabdominal lymph nodes, CNS, bone marrow	Monomorphic lymphoma, Burkitt's, lymphoid granulomatosis, anaplastic large cell	RI, rituximab, surgical resection, CH, XRT	Median survival 10 months
Kremer et al. 2012, [13]	35/705	4.8%	38 months	Lung, GI tract, nasopharynx, skin, CNS, kidney, liver	Polymorphic lymphoma, monomorphic lymphoma	RI, rituximab, CH, XRT	Median survival 18.5 months

*This study examined 6/17 lung transplant patients with PTLD who received rituximab as a first-line therapy.

RI: reduced immunosuppression; CH: chemotherapy, XRT: radiation therapy.
